# Li_2_SnO_3_ as a Cathode Material for Lithium-ion Batteries: Defects, Lithium Ion Diffusion and Dopants

**DOI:** 10.1038/s41598-018-30554-y

**Published:** 2018-08-22

**Authors:** Navaratnarajah Kuganathan, Apostolos Kordatos, Alexander Chroneos

**Affiliations:** 1 Department of Materials, Imperial College London, London, SW7 2AZ United Kingdom; 2 Faculty of Engineering, Environment and Computing, Coventry University, Priory Street, Coventry, CV1 5FB United Kingdom

## Abstract

Tin-based oxide Li_2_SnO_3_ has attracted considerable interest as a promising cathode material for potential use in rechargeable lithium batteries due to its high- capacity. Static atomistic scale simulations are employed to provide insights into the defect chemistry, doping behaviour and lithium diffusion paths in Li_2_SnO_3_. The most favourable intrinsic defect type is Li Frenkel (0.75 eV/defect). The formation of anti-site defect, in which Li and Sn ions exchange their positions is 0.78 eV/defect, very close to the Li Frenkel. The present calculations confirm the cation intermixing found experimentally in Li_2_SnO_3_. Long range lithium diffusion paths *via* vacancy mechanisms were examined and it is confirmed that the lowest activation energy migration path is along the *c-*axis plane with the overall activation energy of 0.61 eV. Subvalent doping by Al on the Sn site is energetically favourable and is proposed to be an efficient way to increase the Li content in Li_2_SnO_3_. The electronic structure calculations show that the introduction of Al will not introduce levels in the band gap.

## Introduction

Lithium ion batteries with high-power densities for electrical vehicles and consumer electronics require high-performance cathode materials providing high concentration of Li^+^ ions in the intercalation/de-intercalation process, low cost, less hazard and their constituent elements being high abundance^1–5^. The search for such cathode materials generated considerable research activity and resulted promising new cathode materials such as Li_2_MSiO_4_ (M = Fe, Mn and Co)^5–13^, Li_2_FePO_4_F^14^, and Li_2_FeP_2_O_7_^15^. Several “Li-rich” materials such as Li_7_Mn(BO_3_)_3_^16^, Li_5_FeO_4_^17,18^, Li_3_V(MoO_4_)_3_^19^ and Li_4_Ti_5_O_12_^20^ have been reported as promising cathode materials producing high theoretical capacity. A key feature of “Li-rich” materials is that, in principle, extraction of more than one lithium per formula unit is possible. Thus, this can produce a higher capacity than the conventional cathode materials such as LiCoO_2_^21^ and LiFePO_4_^22^.

“Li-rich” Li_2_MnO_3_ was recently suggested as a possible alternative cathode material due to its high capacity over 200 m Ahg^–1^ and high energy density^23–25^. However, this material showed poor structural stability during cycling and electronic conductivity. It was suggested that replacement of Mn by Sn could be a possible strategy to improve structural stability and thus cyclic performance of Li_2_MnO_3_^26^. As both Li_2_MnO_3_ and Li_2_SnO_3_ crystalize in a monoclinic layered structure, these studies motivated to consider Li_2_SnO_3_ as a viable cathode material for lithium ion batteries. Recently, Wang *et al*.^27^ have used high-energy X-ray diffraction to determine the structure of Li_2_SnO_3_ and determined that a singnificant amount of intra-layer Li-Sn intermixing is present in the as- prepared material. In another experimental study, Wang *et al*.^28^ have used a hydrothermal route to prepare Li_2_SnO_3_ and observed an electrochemical performance with high capacity and good cycling stability. Howard and Holzworth^29^ have recently studied the Li-ion diffusion mechanism and the lithiation process computationally in both Li_2_SnO_3_ and Li_2_SnS_3_. There are no further theoretical studies availble in the literature detailing the defect processes in this material. A greater insight into the defect properties of electrode materials is crucial to the full understanding of their electrochemical behaviour. Theoretical modelling can bridge this gap by providing detailed information of the key issues related to defect processes including cation mixing observed in experiment and doping strategies to increase the Li concentration in this material.

The present study extends our recent static atomistic simulation studies of the Li_5_FeO_4_^30^ and Li_2_CuO_2_^31^ battery materials where we examined the defect chemistry, lithium transport and dopants. We have carried out a detailed survey of the relative energetics of the formation of intrinsic defects, the solution of dopants, and the possible pathways for lithium ion conduction in Li_2_SnO_3_.

## Results and Discussion

### Li_2_SnO_3_ structure

**Figure 1:**
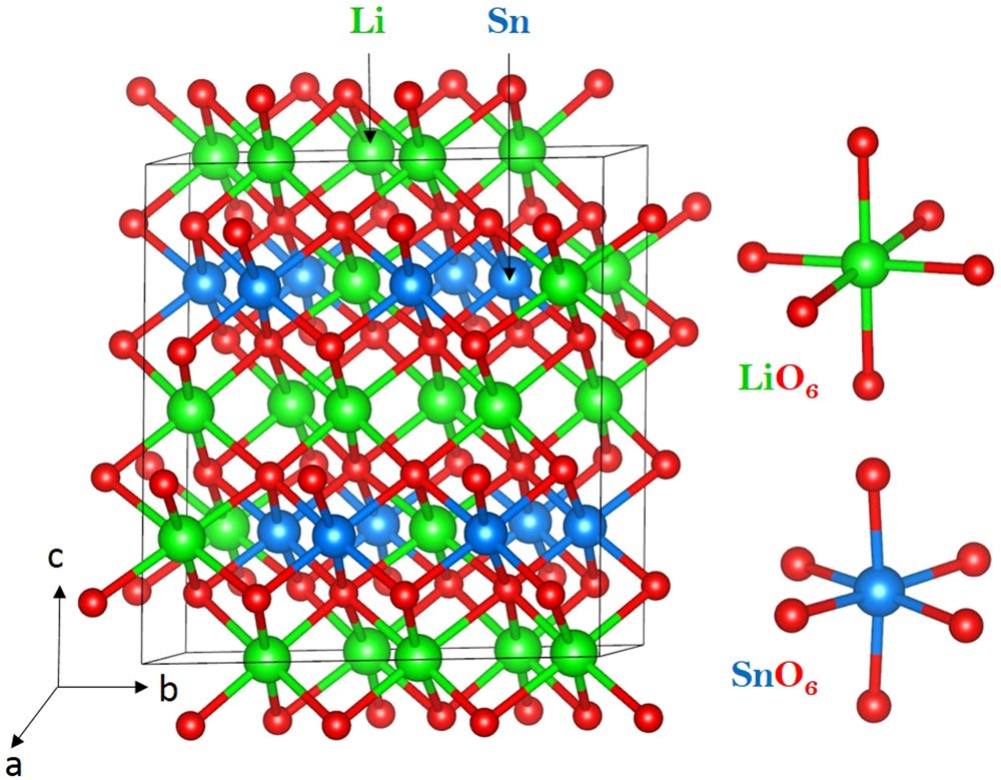
Crystal structure of Li_2_SnO_3_ (space group C2/c).

Crystal structure of Li_2_SnO_3_ exhibits a monoclinic crystallographic structure with space group C2/c (lattice parameters a = 5.290 Å, b = 9.190 Å, c = 10.030 Å, α = 90°, β = 100.1° and γ = 90°) as reported by Lang^32^. Figure 1 shows this structure and the chemical environments of Sn and Li (both forming octahedrons with six O atoms). This material has layers in the *ab* plane with an A-B stacking sequence. The starting point for the present study was to reproduce the experimentally observed monoclinic crystal structure to enable an assessment of the quality and efficacy of the classical pair potentials (refer to Table S1 in the Supplementary Information for the potentials parameters used and method section for the detailed description of the methodology) used in this study. The calculated equilibrium lattice constants (tabulated in Table 1) are in excellent agreement with experiment.

**Table 1:** Calculated structural parameters using classical pair potential method and corresponding experimental values reported for monoclinic (C2/c) Li_2_SnO_3_.

Parameter	Calc	Expt^32^	|∆|(%)
a (Å)	5.3029	5.2900	0.24
b (Å)	9.2563	9.1900	0.72
c (Å)	10.0598	10.0300	0.30
α (°)	90.0	90.0	0.00
β (°)	100.85	100.10	0.75
γ (°)	90.0	90.0	0.00

### Intrinsic defect processes

To gain insights of the electrochemical behavior of an electrode material, we have calculated a series of isolated point defect (vacancy, antisite and interstitial) energies using classical pair potential method and combined them to calculate the formation energies for Frenkel, Schottky and antisite defects in Li_2_SnO_3_. The following equations represent the reactions involving these defects as written using Kröger-Vink notation^27^.

1 $${\rm{Li}}\,{\rm{Frenkel}}:\,{{\rm{Li}}}_{{\rm{Li}}}^{{\rm{X}}}\to {V}_{{\rm{Li}}}^{{\prime} }+{{\rm{Li}}}_{{\rm{i}}}^{\bullet }$$

2 $${\rm{O}}\,{\rm{Frenkel}}:\,{{\rm{O}}}_{{\rm{O}}}^{{\rm{X}}}\to {V}_{{\rm{O}}}^{\bullet \bullet }+{{\rm{O}}}_{{\rm{i}}}^{{\prime} {\prime} }$$

3 $$\mathrm{Sn}\,\,{\rm{Frenkel}}:\,{\mathrm{Sn}}_{{\rm{Sn}}}^{{\rm{X}}}\to \,{V}_{{\rm{Sn}}}{{\prime} {\prime} {\prime} {\prime} }+{\,\mathrm{Sn}}_{{\rm{i}}}^{\bullet \bullet \bullet \bullet }$$

4 $${\rm{Schottky}}:2{{\rm{Li}}}_{\mathrm{Li}\,}^{{\rm{X}}}+{{\rm{Sn}}}_{{\rm{Sn}}}^{{\rm{X}}\,}+3{{\rm{O}}}_{{\rm{O}}}^{{\rm{X}}}\to 2{V}_{{\rm{Li}}}^{{\prime} }+{V}_{{\rm{Sn}}}{{\prime} {\prime} {\prime} {\prime} }+3{V}_{{\rm{O}}}^{\bullet \bullet }+{{\rm{Li}}}_{2}{{\rm{SnO}}}_{3}$$

5 $${{\rm{Li}}}_{2}{\rm{O}}\,{\rm{Schottky}}:2{{\rm{Li}}}_{{\rm{Li}}}^{{\rm{X}}}+{{\rm{O}}}_{{\rm{O}}}^{{\rm{X}}\,}\to 2{V}_{{\rm{Li}}}^{{\prime} }+{V}_{{\rm{O}}}^{\bullet \bullet }+{{\rm{Li}}}_{2}{\rm{O}}$$

6 $${\rm{Li}}/{\rm{Sn}}\,{\rm{antisite}}\,({\rm{isolated}}):{{\rm{Li}}}_{{\rm{Li}}}^{{\rm{X}}}+{\mathrm{Sn}}_{{\rm{Sn}}}^{{\rm{X}}\,}\to {{\rm{Li}}}_{{\rm{Sn}}}^{{\prime} {\prime} {\prime} }+{{\rm{Sn}}}_{{\rm{Li}}}^{\bullet \bullet \bullet }$$

7 $${\rm{Li}}/{\rm{Sn}}\,{\rm{antisite}}\,({\rm{cluster}}):{{\rm{Li}}}_{{\rm{Li}}}^{{\rm{X}}}+{{\rm{Sn}}}_{{\rm{Sn}}}^{{\rm{X}}}\to {\{{{\rm{Li}}}_{{\rm{Sn}}}^{{\prime} {\prime} {\prime} }:{{\rm{Sn}}}_{{\rm{Li}}}^{\bullet \bullet \bullet }\}}^{{\rm{X}}}$$

**Figure 2:**
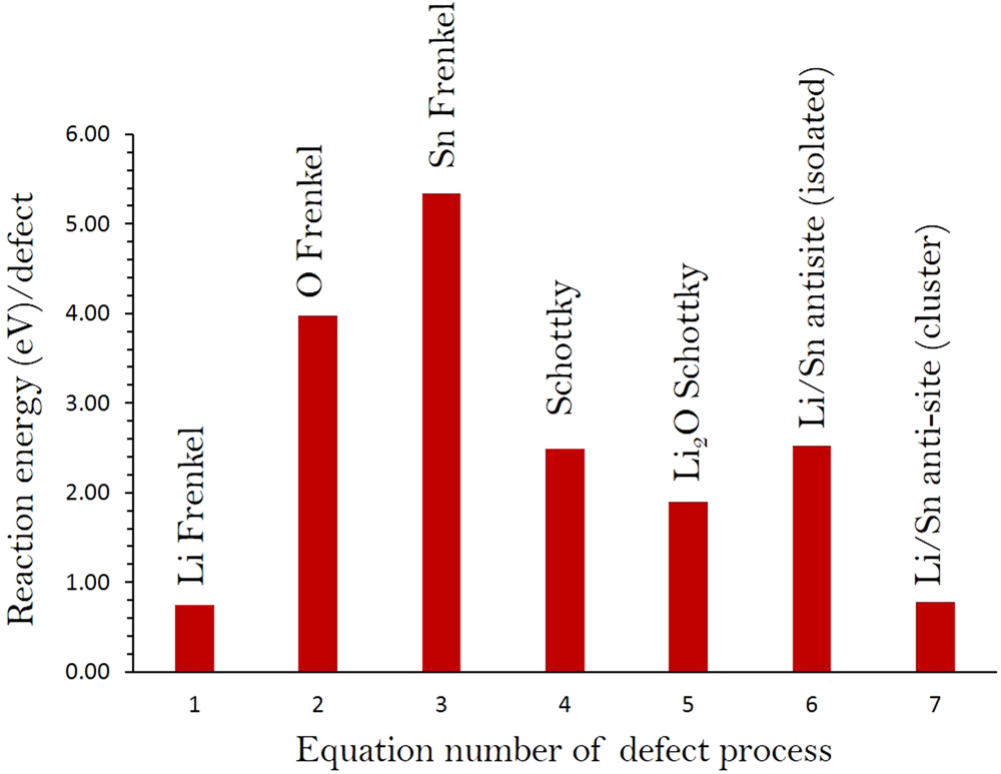
Energetics of intrinsic defect process calculated using classical pair potential method in monoclinic Li_2_SnO_3_.

Figure 2 reports the reaction energies for these intrinsic defect processes. The most favorable intrinsic disorder is Li Frenkel and the formation of other Frenkel and Schottky defects is less favourable. The second most favorable defect process is calculated to be the anti-site indicating that there will be a small percentage of Li on Sn sites (${{\rm{Li}}}_{{\rm{Sn}}}^{{\prime} {\prime} {\prime} }$) and Sn on Li sites (${{\rm{Sn}}}_{{\rm{Li}}}^{\bullet \bullet \bullet })$ particularly at higher temperatures. This is in agreement with the intralayer intermixing between Li and Sn found in the experiment^27^. Antisite defects have been observed in a variety of other Li ion battery materials during cycling^8,33–37^. The formation enthalpy of Li_2_O via the Li_2_O Schottky-like reaction (relation 5) is a processes that requires an energy of 1.90 eV per defect (refer to Table S2). This is a process that can lead to further $\,{V}_{Li}^{{\prime} }$ and ${V}_{O}^{\bullet \bullet }\,\,$but at elevated temperatures.

### Lithium ion-diffusion

**Figure 3:**
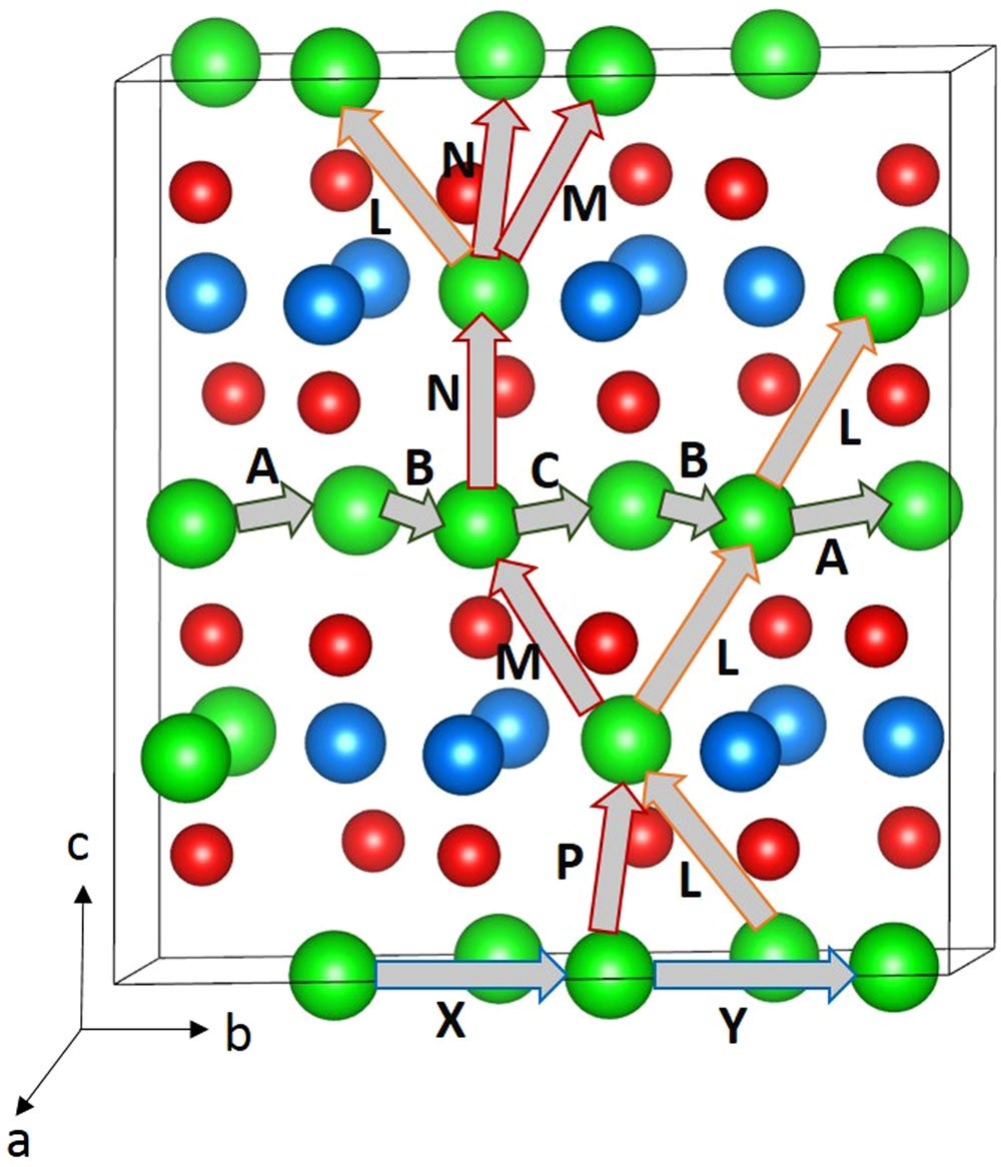
Possible long range lithium vacancy migration paths considered. Green, blue and red colors correspond to Li, Sn, and O atoms respectively.

**Figure 4:**
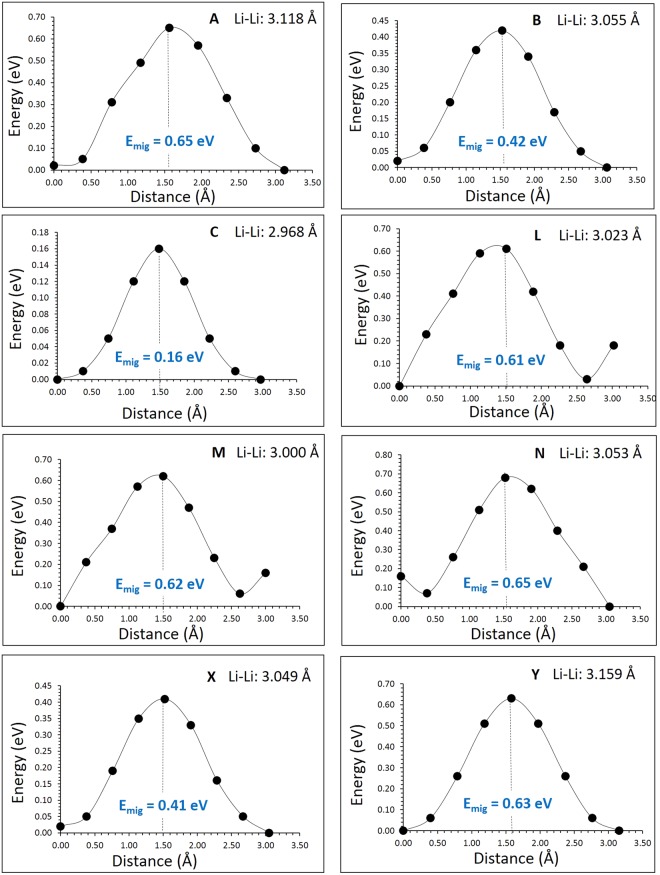
Eight different energy profiles [as shown in Fig. 3] of Li vacancy hopping between two adjacent Li sites in Li_2_SnO_3_.

Promising high-rate cathode materials in lithium ion batteries require lithium ion diffusion with lower activation energy. Using classical pair potential method it is possible to examine various possible diffusion paths responsible for lithium ion diffusion and they provide experiment with complementary information. For the Li vacancy migration, we have calculated eight different local Li hops (refer to Fig. 3). Migration energies are reported in Table 2 together with the Li-Li separation, whereas energy profile diagrams are shown in Fig. 4. We have constructed long range paths connecting local Li hops with lower overall activation energy. We have identified two long range paths along the *ab* plane (refer to Fig. 3). The first long range path exhibits a zig-zag pattern (A → B → C → B → ttincluding a local Li hop with lower activation energy of migration of 0.16 eV (local hop C) but with overall activation energy of 0.65 eV (refer to Table 2). The second path exhibits a straight line (X → Y) with an activation energy of 0.63 eV. There are different possible long range Li diffusion paths can be constructed along the c-axis. Our examination reveals that the lower activation energy long range path along the c-axis plane is L → L → L (refer to Fig. 3) with overall activation energy of 0.61 eV. Other long range paths will have activation energies greater than 0.61 eV in this direction as their local Li hops show higher energies. The activation energy of Li-vacancy migration calculated along the c axis is reported to be 0.30 eV by Howard and Holzwarth^29^, which differs from our calculated value of 0.61 eV. The difference in activation energy can be due to difference in the *c*-lattice parameter calculated in their calculation (9.78 Å) and our calculation (10.059 Å). The experimental *c*-lattice parameter is reported to be 10.03 Å^32^. Here the activation energy of migration is defined as the position of the highest potential energy along the migration path.

**Table 2:** Calculated Li-Li separations and activation energies using classical pair potential method for the lithium ion migration between two adjacent Li sites refer to Fig. 3.

Migration path	Li-Li separation (Å)	Activation energy (eV)
A	3.118	0.65
B	3.055	0.42
C	2.968	0.16
L	3.023	0.61
M	3.000	0.62
N	3.053	0.65
X	3.049	0.41
Y	3.159	0.63

### Trivalent doping

Incorporation of extra lithium into the as-prepared material will enhance the capacity and further increase the applicability of Li_2_SnO_3_ as a cathode material for rechargeable lithium batteries. A possible approach to increase the amount of lithium is by doping trivalent cations on Sn site through creating Li interstitials. Similar approach has been previously demonstrated in Li_2_MnSiO_4_ cathode material^12^. Here we considered the solution of ${R}_{2}{O}_{3}$ (*R* = Al, Sc, In, Y, Gd and La) via the following process (in Kröger-Vink notation):

8 $${{\rm{R}}}_{2}{{\rm{O}}}_{3}+2{{\rm{Sn}}}_{{\rm{Sn}}}^{{\rm{X}}}+{{\rm{Li}}}_{2}{\rm{O}}\to 2{{\rm{R}}}_{{\rm{Sn}}}^{{\prime} }+2{{\rm{Li}}}_{{\rm{i}}}^{\bullet }+2{{\rm{SnO}}}_{2}$$

**Figure 5:**
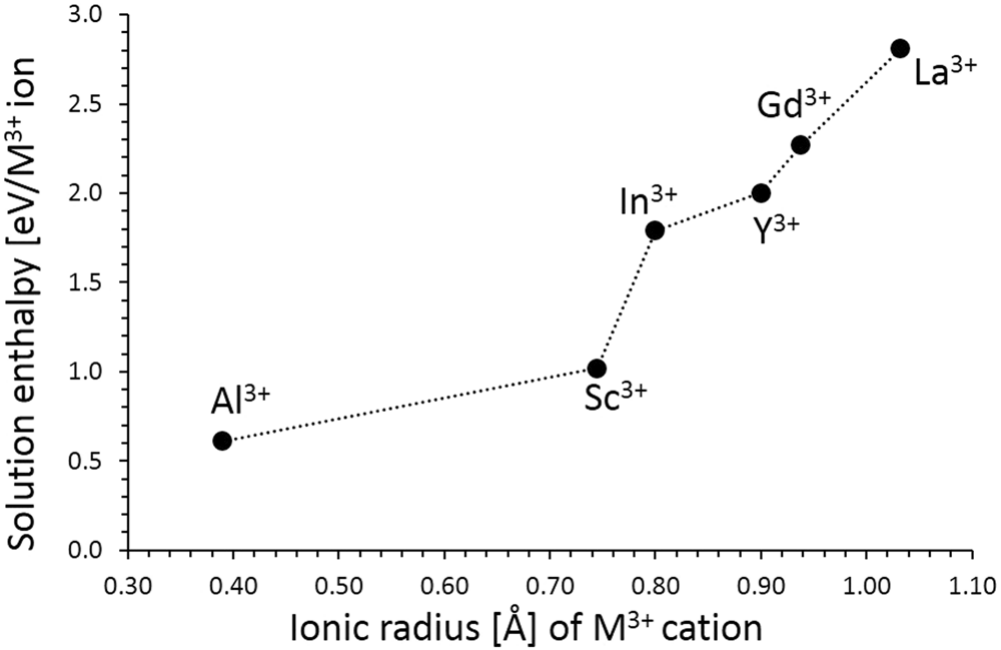
Enthalpy of solution of ${{\rm{R}}}_{2}{{\rm{O}}}_{3}$ (*R* = Al, Sc, In, Y, Gd and La) with respect to the R^3+^ ionic radius in Li_2_SnO_3_.

Figure 5 reports the solution energies of ${R}_{2}{O}_{3}\,\,$calculated using classical pair potential method and it can be observed that the most favorable dopant solution energy (0.60 eV) is for Al^3+^. This suggests a possible synthesis-doping strategy to introduce additional lithium into Li_2_SnO_3_, although the exact amount of Al incorporation cannot be predicted. The possible composition of Al-doped Li_2_SnO_3_ would be Li_2+x_Sn_1−x_Al_x_O_3_ (x = 0.0–1.0). The second most favorable dopant is Sc^3+^ with the solution energy of 1.02 eV. The solution energy increases further with the dopant size.

**Figure 6:**
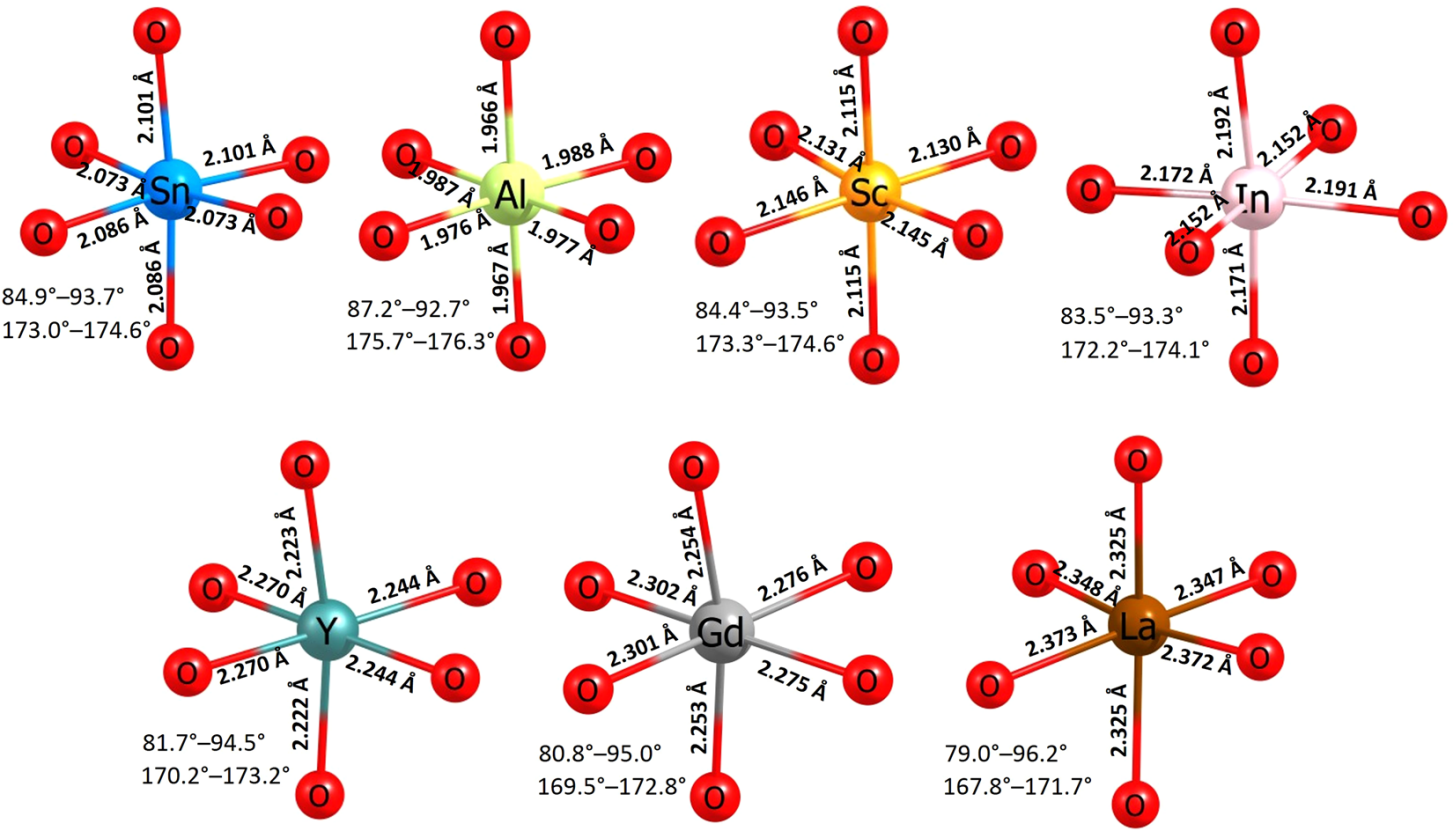
Octahedral SnO_6_ unit in the relaxed structure of undoped Li_2_SnO_3_ and the coordination formed by the dopants on the Sn site with neighbor oxygen.

Figure 6 depicts the local coordination (including bond lengths and angles) with oxygen of the dopants occupying the Sn site and for comparison the octahedral SnO_6_ unit in the relaxed structure of undoped Li_2_SnO_3_. The ionic radius of Sn^4+^ in octahedral coordination is 0.69 Å. The ionic radius of Al^3+^ is 0.16 Å smaller than that of Sn^4+^. In the AlO_6_ unit, all six Al-O bonds are shorter compared to the Sn-O bonds present in the undoped Li_2_SnO_3_ and the other R-O bonds. This is due to its smaller cation size of Al^3+^ which strongly polarises the oxygen ions forming strong ionic bonds with O atoms. The second lowest solution energy is found for Sc^3+^. Its ionic radius is 0.05 Å and 0.20 Å longer than the Sn^4+^ and Al^3+^ respectively. Bigger size of the Sc^3+^compared to Al^3+^ shows a higher solution energy. From In to La, dopant-oxygen bond distances increase and bond angles decrease gradually indicating the structural distortion and reflecting in the solution energies. In the relaxed structure of LaO_6_ unit, La–O bond lengths are approximately the same but ~0.30 Å longer than Sn–O bond lengths present in SnO_6_ unit. Furthermore, the ionic radius of La^3+^ is 0.35 Å greater than Sn^4+^. Thus the solution energy is high. However, the current solution energy values are large and positive indicating that they are unfavorable.

### Densities of states

**Figure 7:**
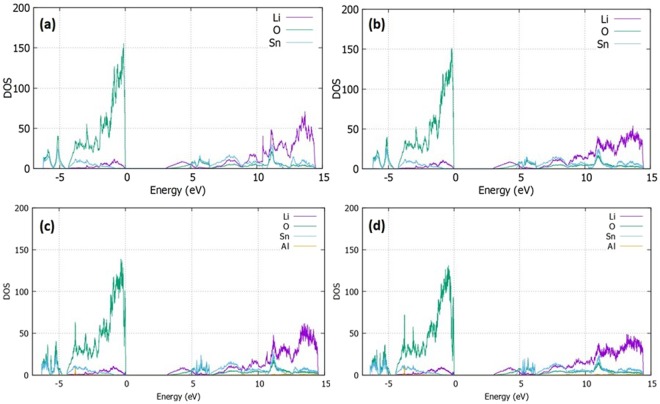
The PDOS calculated using DFT for the (**a**) Li_2_SnO_3_ perfect cell (**b**) Li_2_SnO_3_ with Li interstitial (**c**) Al – Doped Li_2_SnO_3_ and (**d**) Al- Doped Li_2_SnO_3_ with Li interstitial.

For the trivalent dopants considered we have used density functional theory (DFT) and have performed density of states (DOS) calculations plotting the individual contribution of each element (partial-DOS). In Fig. 7, the PDOS for the cases of the (a) perfect cell (b) ${{\rm{Li}}}_{{\rm{i}}}^{\bullet }$ (c) ${{\rm{Al}}}_{{\rm{Sn}}}^{{\prime} }$ and (d) $\{{{\rm{Al}}}_{{\rm{Sn}}}^{{\prime} }:{{\rm{Li}}}_{{\rm{i}}}^{\bullet }$}^X^ are reported. The valence band maximum (VBM) is set at zero energy level. The electronic structure is characterized by the dominant O^2−^ p-states in the valence band near the Fermi level whereas a band gap of approx. 3.1 eV is formed. The Li^+^ p-states and Sn^4+^ d-states are also contributing in the valence bands but with lower intensities as compared to O^2−^. In the edge of the conduction band, the main contributions in the total DOS are attributed to the Li states and specifically to the Li^+^ - s orbitals. The O^2−^ p-states in the conduction band correspond to a similar profile as the Li^+^ p–states whereas the Sn^4+^ orbitals are presented not to have a primary role in the total electronic structure of the material (refer also to Figure S1 in the Supplementary Information). In addition, the effect on the total DOS with the introduction of a lithium interstitial in the supercell is minimal. Specifically, no additional states are formed into the band gap, whereas the contribution of every element is not affected as shown in Fig. 7(b). The doping effect of trivalent dopants have been also considered and the impact of the $A{l}_{Sn}^{\bullet }\,\,$and $\{A{l}_{Sn}^{\bullet }:\,L{i}_{i}^{\bullet }$}^*X*^ pairs on the densities of states on Li_2_SnO_3_ is presented in Fig. 7(c,d). Overall, the introduction of Al^3+^ does not impact significantly the electronic structure as the Al – states contribution in the DOS is weak. Furthermore, the cases of $S{c}_{Sn}^{\bullet }\,\,$and $\{S{c}_{Sn}^{\bullet }:L{i}_{i}^{\bullet }$}^*X*^, $I{n}_{Sn}^{\bullet }\,\,$and $\{I{n}_{Sn}^{\bullet }:L{i}_{i}^{\bullet }$}^*X*^, ${Y}_{Sn}^{\bullet }\,\,$and $\{{Y}_{Sn}^{\bullet }:L{i}_{i}^{\bullet }$}^*X*^, $G{d}_{Sn}^{\bullet }\,\,$and $\{G{d}_{Sn}^{\bullet }:L{i}_{i}^{\bullet }$}^*X*^ as well as $L{a}_{Sn}^{\bullet }\,\,$and $\{L{a}_{Sn}^{\bullet }:L{i}_{i}^{\bullet }$}^*X*^ pairs have been also examined (Please refer to Figure S2 in the Supplementary Information). Despite the fact of the dopants having stronger contributions as compared with Al^3+^, the electronic structure presents no considerable variations. However, for the $G{d}_{Sn}^{\bullet }\,\,$and $\{G{d}_{Sn}^{\bullet }:L{i}_{i}^{\bullet }$}^*X*^ as well as the $L{a}_{Sn}^{\bullet }\,\,$and the $\{L{a}_{Sn}^{\bullet }:L{i}_{i}^{\bullet }$}^*X*^ pairs that correspond to the dopants of a large ionic radius, the DOS includes in-gap states (Fig. S2 (g,h)) and a minor additional contribution next to the valence band (Fig. S2 (i,j)).

### Summary

In the present study, we have used atomistic simulation techniques to provide detailed insights into intrinsic defects, lithium ion mobility and trivalent doping, which are relevant to the general electrochemical behaviour of layered Li_2_SnO_3_ as a lithium battery cathode material. The Li Frenkel is the lowest energy and thus the dominant defect energy process. Anti-site disorder is only 0.03 eV higher than the Li Frenkel suggesting that there will be some Sn on the Li site and vice-versa and our calculation confirms this defect that has been experimentally observed. Considering the vacancy mechanism of diffusion the lowest activation energy migration path is along the *c-*axis plane with an activation energy of 0.61 eV. We have considered the solution energies of ${R}_{2}{O}_{3}$ (*R* = Al, Sc, In, Y, Gd and La) to create extra lithium in this material and found that Al_2_O_3_ have the lowest solution energy and Al defects do not introduce levels in the band gap. The present study motivates experimental work on Al-doped Li_2_SnO_3_ as this is expected to be an important lithium ion battery material.

### Methods

In order to calculate the energetics for the formation of intrinsic defects and possible Li ion diffusion pathways, the classical pair potential method as implemented in the GULP package^38^ was employed. This method is based on the classical Born model description of an ionic crystal lattice. All systems were treated as crystalline solids with interactions between ions consisting of the long-range attractions and short-range repulsive forces representing electron-electron repulsion and van der Waals interactions. The short range interactions were modelled using Buckingham potentials (refer to Table S1). Simulation boxes and the corresponding atom positions were relaxed using the Broyden-Fletcher-Goldfarb-Shanno (BFGS) algorithm^39^. The Mott-Littleton method^40^ was used to investigate the lattice relaxation about point defects and the migrating ions. It divides the crystal lattice into two concentric spherical regions, where the ions within the inner spherical region (on the order of >700 ions) immediately surrounding the defect relaxed explicitly. Li ion diffusion was calculated considering two adjacent vacancy sites as initial and final configurations. Seven interstitial Li ions were considered in a direct linear route and they were fixed while all other ions were free to relax. The local maximum energy along this diffusion path is calculated and reported as activation energy of migration. As the present model assumes a full charge ionic model with the calculations corresponding to the dilute limit the defect enthalpies will be overestimated, however, relative energies and trends will be consistent.

The Li_2_SnO_3_ supercells were modelled though the plane wave density functional theory code CASTEP^41,42^. The plane wave basis set was set to a cut-off of 480 eV and a 2 × 2 × 2 Monkhorst-Pack (MP)^43^ k-point grid was used with a 98-atomic site supercell. For the exchange and correlation interactions in the crystal, we use the formulation with the corrected density functional of Perdew, Burke and Ernzerhof (PBE)^44^, within the generalized gradient approximation (GGA) with ultrasoft pseudopotentials^45^. All the calculations were under constant pressure conditions and the cells were relaxed in the minimum energy configuration before the investigation of the electronic structure. The Partial Density of States (PDOS) for the perfect and defective/doped structures is visualized through the OPTADOS tool^46,47^.

## Electronic supplementary material

Supplementary Information
