# Supplementary material for: Li2SnO3 as a Cathode Material for Lithium-ion Batteries: Defects, Lithium Ion Diffusion and Dopants
Source: Sci Rep. 2018 Aug 22;8:12621. doi: 10.1038/s41598-018-30554-y (PMC6105723; doi:10.1038/s41598-018-30554-y)
Supplement: Supplementary file 1 — Supplementary Information [file 41598_2018_30554_MOESM1_ESM.docx]

**Supporting Information**

**Li_2_SnO_3_ as a Cathode Material for Lithium-ion Batteries: Defects, Lithium Ion Diffusion and Dopants**

Navaratnarajah Kuganathan^1^, ApostolosKordatos,^2^ and Alexander Chroneos^1,2^

*^1^Department of Materials, Imperial College London, London, SW7 2AZ, United Kingdom2*

*^3^Faculty of Engineering, Environment and Computing, Coventry University, Priory Street, Coventry CV1 5FB, United Kingdom*

Corresponding authors, e-mails: a) n.kuganathan@imperial.ac.uk

b) [alexander.chroneos@imperial.ac.uk](mailto:alexander.chroneos@imperial.ac.uk)

**Table S1**. Interatomic potential parameters used in the atomistic simulations of Li_2_SnO_3_.

Two-body [Φ*_ij_* (*r_ij_*) = *A_ij_* exp (− *r_ij_* /*ρ_ij_*) − *C_ij_ / r_ij_*^6^]

| Interaction | *A* (eV) | *ρ* (Å) | *C* (eV·Å^6^) | Y (e) | K (eV·Å^-2^) |
| --- | --- | --- | --- | --- | --- |
| Li^+^–O^2−^ | 632.1018 | 0.2906 | 0.00 | 1.000 | 99999 |
| Sn^4+^–O^2−^ | 1414.32 | 0.3479 | 13.66 | 4.000 | 99999 |
| O^2−^–O^2−^ | 22764.30 | 0.1490 | 27.627 | –2.75823 | 30.211 |
| Al^3+^ - O^2−^ | 1725.20 | 0.28971 | 0.000 | 3.000 | 99999 |
| Sc^3+^ - O^2−^ | 1575.85 | 0.3211 | 0.000 | 3.000 | 99999 |
| In^3+^ - O^2−^ | 1495.65 | 0.3327 | 4.33 | 3.000 | 99999 |
| Y^3+^ - O^2−^ | 1766.40 | 0.33849 | 19.43 | 3.000 | 99999 |
| Gd^3+^ - O^2−^ | 1885.75 | 0.3399 | 20.34 | 3.000 | 99999 |
| La^3+^ - O^2−^ | 2088.79 | 0.3460 | 23.25 | 3.000 | 99999 |

**Table S2****.** Energetics of intrinsic defect process in Li_2_SnO_3_

| Defect process/equation | Reaction energy/eV | Reaction energy per defect/eV |
| --- | --- | --- |
| Li Frenkel /1 | 1.50 | 0.75 |
| O Frenkel /2 | 7.96 | 3.98 |
| Sn Frenkel /3 | 10.68 | 5.34 |
| Schottky /4 | 14.96 | 2.49 |
| Li_2_O Schottky/5 | 5.71 | 1.90 |
| Li/Sn antisite (isolated) /6 | 5.04 | 2.52 |
| Li/Sn antisite (cluster) /7 | 1.56 | 0.78 |


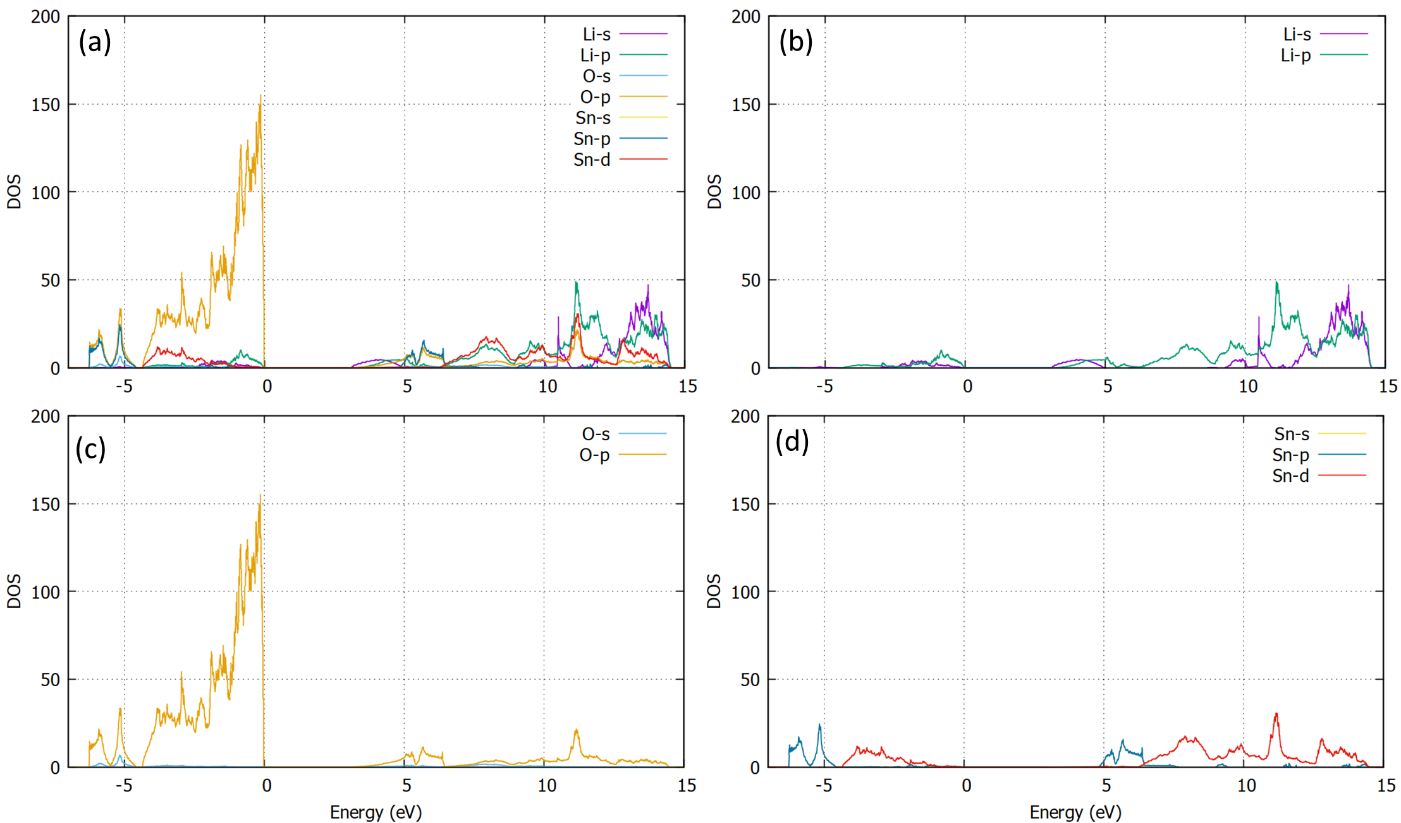


**Figure 1. (a)** The complete contribution of the atomic orbitals in the Densities of States for the undoped Li_2_SnO_3_ **(b)** The contribution of Li orbitals **(c)** The contribution of O orbitals and **(d)** The contribution of Sn orbitals


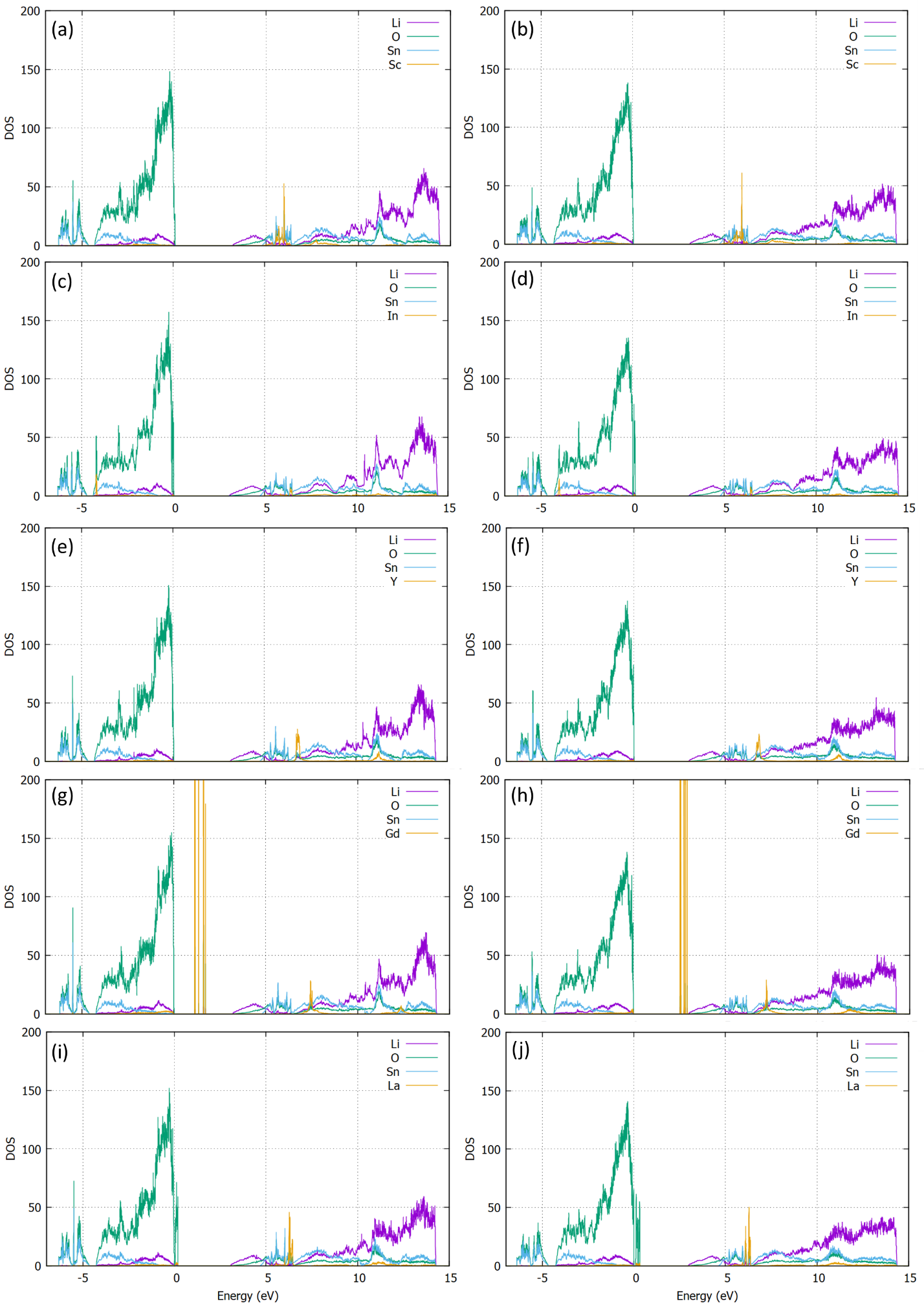


**Figure 2.** The Li_2_SnO_3_ Densities of States for **(a)** Sc – doped supercell **(b)** Sc – doped supercell with one Li interstitial **(c)** In – doped supercell **(d)** In – doped supercell with one Li interstitial **(e)** Y – doped supercell **(f)** Y – doped supercell with one Li interstitial **(g)** Gd – doped supercell **(h)** Gd – doped supercell with one Li interstitial **(i)** La – doped supercell **(j)** La – doped supercell with one Li interstitial
